# Environmental drivers and ecological risks of bisphenol A occurrence in Chao Phraya River, Thailand

**DOI:** 10.1016/j.isci.2026.117460

**Published:** 2026-09-07

**Authors:** Sarima Niampradit, Wissanupong Kliengchuay, Rachaneekorn Mingkhwan, Nuttapohn Kiangkoo, Tret C. Burdette, Kunrunya Sutan, Yanin Limpananont, Suwalee Worakhunpiset, Surat Hongsibsong, Walaiporn Phonphan, Parinya Panuwet, Kyungho Choi, Duangrat Inthorn, Kraichat Tantrakarnapa

**Affiliations:** 1 Department of Social and Environmental Medicine, Faculty of Tropical Medicine, Mahidol University, Bangkok 10400, Thailand; 2 Environment, Health & Social Impact Unit, Faculty of Tropical Medicine, Mahidol University, Bangkok 10400, Thailand; 3 Center for Applied Isotope Studies, University of Georgia, Athens, GA 30602, USA; 4 Environmental and Occupational Health Sciences Unit, Research Institute for Health Sciences, Chiang Mai University, Chiang Mai 50200, Thailand; 5 School of Health Sciences Research, Research Institute for Health Sciences, Chiang Mai University, Chiang Mai 50200, Thailand; 6 Faculty of Science and Technology, Suan Sunandha Rajabhat University, Bangkok, Thailand; 7 Division of Environmental Laboratory Science, Department of Environmental Protection, Lawrence, MA 01810, USA; 8 Gangarosa Department of Environmental Health, Rollins School of Public Health, Emory University, Atlanta, GA 30328, USA; 9 Graduate School of Public Health, Seoul National University, Gwanak-gu, Seoul 08826, South Korea; 10 Faculty of Environment and Resource Studies, Mahidol University, Nakhon Pathom 73170, Thailand

**Keywords:** bisphenol A, Chao Phraya River, land use analysis, ecological risk assessment, tropical river system

## Abstract

Bisphenol A (BPA) is a ubiquitous industrial chemical, yet its behavior in tropical river systems remains poorly understood. This study presents the first integrated assessment of BPA occurrence, controlling factors, and ecological risks in the Chao Phraya River, Thailand. Surface water samples collected in October 2023 were analyzed using ultra-performance liquid chromatography with a quadrupole time-of-flight mass spectrometer (UPLC-QTOF-MS). BPA was detected at all sites, with concentrations ranging from 21.61 to 113.07 ng/L (mean: 60.44 ng/L), exhibiting a clear downstream increase. Multivariate analyses revealed a dual-control mechanism governing BPA distribution: (1) an urban-industrial gradient representing source inputs and (2) a water quality axis, where dissolved oxygen (DO) was the strongest predictor (β = −22.90, *p* < 0.001), indicating that low-oxygen conditions enhance BPA persistence. Ecological risk assessment indicated negligible acute risk but widespread high chronic risk. Human health risk assessment showed negligible non-carcinogenic risk for adults and children, underscoring wastewater management, river oxygenation, and routine BPA monitoring programs.

## Highlights

• Integrated assessment of BPA occurrence and ecological risks in a tropical river
• Low dissolved oxygen and urban inputs influence BPA occurrence
• High chronic ecological risk supports routine BPA monitoring
• Wastewater management and river oxygenation may reduce BPA contamination

## Graphical abstract

**Figure:**
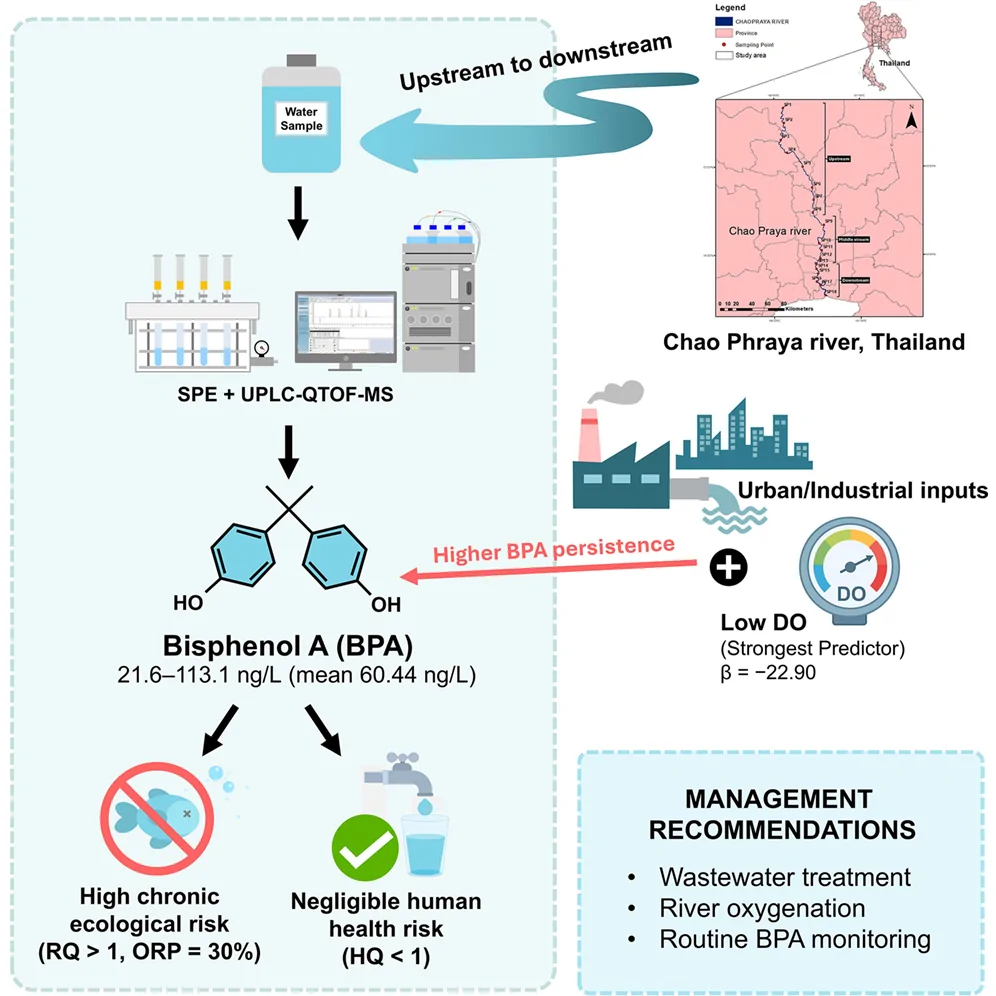

## Teaser

Analytical chemistry applications; Environmental health; Risk assessment

## Introduction

Bisphenol A (BPA), chemically known as 4,4′-isopropylidenediphenol, is one of the most extensively used industrial chemicals globally. It serves as a monomer in the production of epoxy resins and polycarbonate plastics, as well as an additive in polyvinyl chloride and water pipes.^1^ Due to its extensive applications in packaging, electronics, construction materials, and consumer products, the global demand for BPA continues to rise, reaching a market value of approximately USD 20.95 billion in 2023 and projected to grow to USD 35.59 billion by 2028.^2^ However, this widespread use and inadequate control of industrial and municipal discharges have resulted in its frequent detection in aquatic environments worldwide, raising major environmental and ecological concerns.^2^^,^^3^^,^^4^

BPA is classified as an endocrine-disrupting chemical (EDC) because of its ability to mimic endogenous hormones and interfere with normal endocrine function in both humans and aquatic organisms.^5^^,^^6^ Although BPA shows moderate water solubility (120–300 mg/L at 25°C) and a relatively low potential for bioaccumulation (log Kow = 3.64), its continuous release into aquatic systems and long-term persistence make it a contaminant of emerging global concern.^7^ Numerous studies have shown that BPA induces estrogenic responses, oxidative stress, impaired reproduction, and developmental abnormalities in a wide range of aquatic organisms, including algae, crustaceans, and fish.^8^^,^^9^^,^^10^ Even at nanogram per liter concentrations, chronic exposure can disrupt growth, fertility, and metabolic processes,^11^^,^^12^ emphasizing its long-term ecological significance.

Although wastewater treatment plants (WWTPs) can remove a substantial portion of BPA through biological degradation and sorption processes, reported removal efficiencies in conventional systems typically range from 60% to 90%.^13^^,^^14^ Notably, a significant fraction of this apparent removal occurs through adsorption onto sewage sludge rather than complete degradation, leading to its persistence in both effluents and biosolids.^7^^,^^14^ With global effluent concentrations ranging from 0.23 to 149 μg/L, residual BPA consistently enters receiving waters.^15^^,^^16^ In Southeast Asia, where rivers are heavily relied upon for domestic and industrial use, incomplete removal of BPA and other organic pollutants is widespread.^17^^,^^18^^,^^19^ BPA concentrations in Malaysian rivers have been reported from 1.3 to 215 ng/L,^19^ while concentrations as high as 34.97–1554.14 ng/L were found in Thailand’s Nan River, largely due to untreated municipal wastewater.^20^ These findings highlight the burden of BPA contamination in rapidly developing tropical regions.

Anthropogenic pressures strongly influence BPA occurrence and distribution in surface waters. Urban expansion, industrial discharges, agricultural runoff, landfill leachates, and stormwater inputs are recognized as major sources of BPA pollution.^4^^,^^21^^,^^22^ In highly urbanized catchments, such as the Qinhuai River Basin in China, wastewater discharges and urban runoff have been identified as dominant contributors to bisphenol contamination.^23^ Industrial sectors related to plastics, metal finishing, electronics, and textile manufacturing further elevate BPA levels and drive spatial variability in riverine systems.^2^^,^^24^ Land-use transformations from agricultural or forested land to urban and industrial zones have been associated with higher BPA concentrations, indicating that watershed characteristics play a crucial role in pollutant dynamics.^25^

The fate and behavior of BPA in aquatic systems are highly sensitive to the unique environmental conditions of tropical rivers. In warm tropical waters, higher temperatures can enhance the solubility and desorption of BPA from sediments back into the water column, while simultaneously influencing its biodegradation rates.^26^^,^^27^ Furthermore, in highly turbid river systems, BPA’s moderate hydrophobicity facilitates its partitioning onto suspended colloidal particles and sediments, which serve as mobile carriers for pollutant transport.^28^^,^^29^ Under conditions of low dissolved oxygen (DO), which are common in urbanized tropical reaches, the aerobic microbial degradation of BPA may be significantly impeded, leading to increased persistence of the compound in the water.^27^ Understanding these interactions is critical, as the combination of high temperature, high turbidity, and low DO, which are typical of rapidly urbanizing tropical basins, may exacerbate the ecological risks posed by BPA compared to temperate systems.

The Chao Phraya River Basin, Thailand’s largest and most economically vital watershed, receives wastewater from diverse sources, including industrial estates, agricultural zones, and the Bangkok metropolitan area.^30^ Despite its environmental importance, the factors driving BPA contamination along the river’s longitudinal gradient remain poorly understood. A clearer understanding of how land-use patterns and water-quality conditions influence BPA distribution is essential for improving pollution control and safeguarding aquatic ecosystems.

Therefore, this study aimed to address these knowledge gaps by investigating the occurrence, controlling factors, and ecological risks of BPA in the Chao Phraya River. The specific objectives were (1) to determine the occurrence and concentration levels of BPA in surface water, (2) to identify key environmental and anthropogenic drivers using integrated water quality and land-use data through principal component analysis (PCA) and multiple linear regression (MLR), and (3) to assess ecological risks using both deterministic (risk quotient [RQ] based on species sensitivity distributions [SSD]) and probabilistic (joint probability curve [JPC]) approaches. This study provides novel insight into the combined role of source inputs and environmental persistence in controlling BPA dynamics in a tropical river system.

## Results and discussion

### Occurrence of BPA in surface water of Chao Phraya River

Physicochemical parameters of surface water samples were summarized in Table 1. DO ranged from 1.87 to 6.67 mg/L, exhibiting a significant declining trend from upstream to downstream. This depletion in the lower reaches is closely linked to the high influx of organic matter from densely populated urban areas (43% of land use) and aquaculture activities (21%), which accelerate microbial respiration and biochemical oxygen demand.^31^ The pH remained near neutral (6.77–7.50), reflecting the natural buffering capacity of the river system. Electrical conductivity (EC) significantly increased from the middle reaches to the river mouth, peaking at 7821 μs/cm. This sharp rise is primarily attributed to saltwater intrusion, which is strongly governed by seasonal water-level fluctuations and the discharge volume adjusted at the Chao Phraya Dam.^32^ Turbidity levels ranged from 107 to 272 Nephelometric Turbidity Unit (NTU), with higher values in the upstream area. This variability is driven by suspended particles from land-surface erosion and agricultural runoff, reflecting the relative contamination from human activities and land-use patterns along the riverbanks.^33^

**Table 1:** Sampling locations and physicochemical characteristics of surface water collected from the Chao Phraya River

Sample name	Province	Location	DO (mg/L)	pH	EC (μs/cm)	Turbidity (NTU)

**Upstream**						

SP1	Nakhon Sawan	15°40′05.2″*N* 100°06′23.3″E	4.43	7.22	439	248
SP2	Nakhon Sawan	15°30′03.1″*N* 100°07′00.7″E	4.37	6.77	462	201
SP3	Chainat	15°18′31.1″*N* 100°04′52.4″E	4.33	7.50	462	272
SP4	Chainat	15°09′42.8″*N* 100°09′13.9″E	4.09	7.36	482	153
SP5	Singburi	15°00′17.9″*N* 100°19′54.5″E	6.05	6.99	513	135
SP6	Singburi	14°45′02.9″*N* 100°26′53.3″E	6.67	6.93	537	136
SP7	Angthong	14°37′45.5″*N* 100°27′36.4″E	4.63	6.99	436	261
SP8	Angthong	14°29′00.6″*N* 100°26′58.0″E	4.65	6.94	435	255
–	–	**average**	**4.90**	**7.09**	**471**	**208**

**Middle stream**						

SP9	Phra Nakhon Si Ayutthaya	14°20′37.4″*N* 100°34′35.2″E	2.78	6.97	678	142
SP10	Phra Nakhon Si Ayutthaya	14°07′46.0″*N* 100°31′23.5″E	3.97	6.95	555	129
SP11	Pathumthani	14°03′15.0″*N* 100°32′58.9″E	3.74	7.07	563	114
SP12	Pathumthani	13°58′01.0″*N* 100°31′58.2″E	3.46	7.17	580	107
SP13	Nonthaburi	13°53′50.9″*N* 100°29′21.7″E	3.23	6.93	604	158
–	–	**average**	**3.44**	**7.02**	**596**	**130**

**Downstream**						

SP14	Nonthaburi	13°50′31.1″*N* 100°29′23.2″E	3.18	7.11	591	152
SP15	Bangkok	13°47′17.1″*N* 100°30′26.8″E	3.01	6.87	305	125
SP16	Bangkok	13°41′58.0″*N* 100°29′24.6″E	2.70	7.04	669	122
SP17	Samut Prakan	13°39′33.7″*N* 100°32′09.6″E	1.94	7.27	692	117
SP18	Samut Prakan	13°32′38.2″*N* 100°35′18.1″E	1.87	7.24	7821	142
–	–	**average**	**2.54**	**7.11**	**2016**	**131**

**Figure 1:**
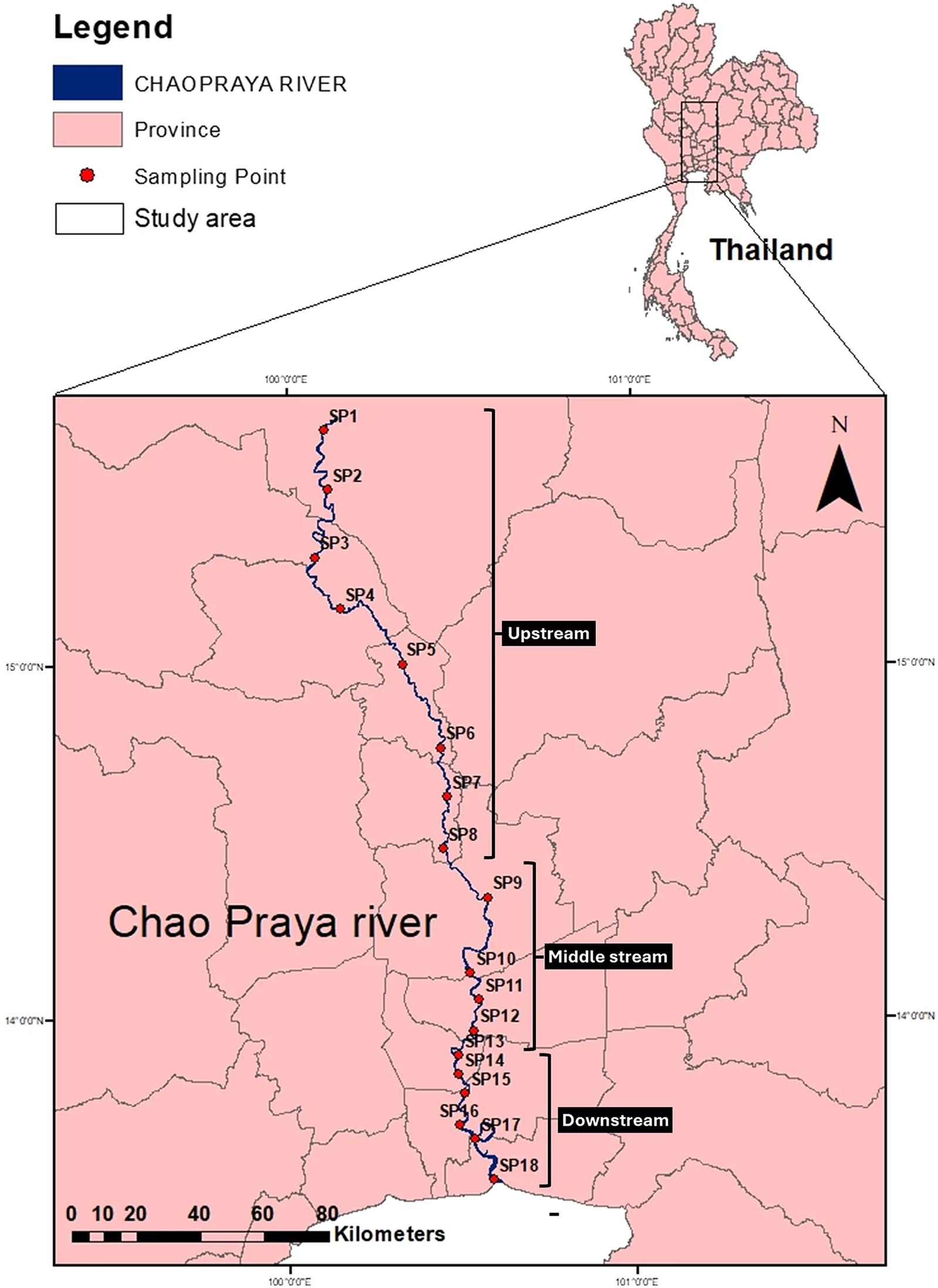
Sampling location along Chao Phraya River for bisphenol A analysis Scale bars, 80 km.

**Figure 2:**
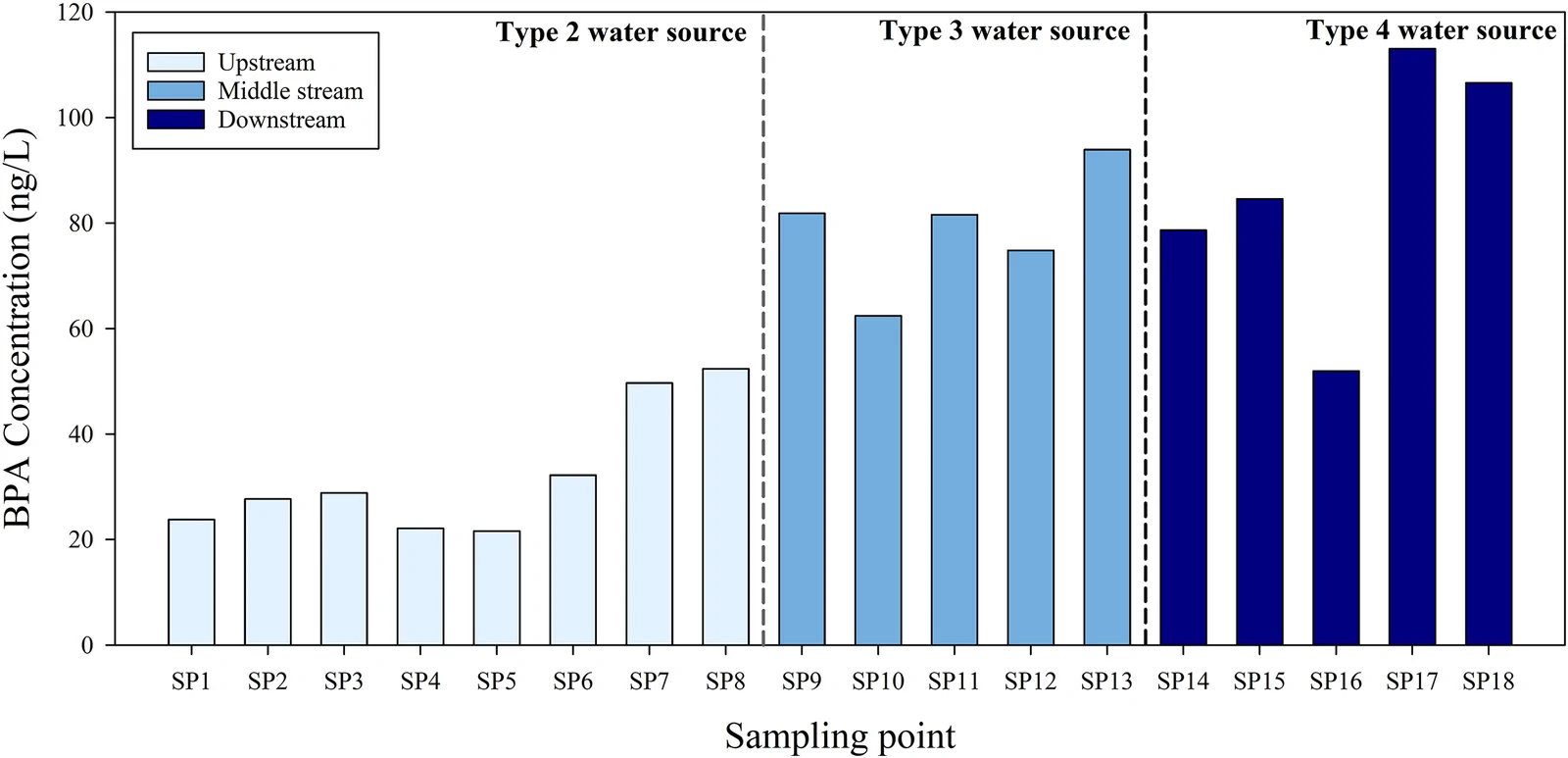
Concentration of bisphenol A in surface water of Chao Phraya River

BPA was detected in all samples collected from the sampling points (SPs) along the Chao Phraya River (Figure 1), with an average concentration of 60.44 ng/L, ranging from 21.61 to 113.07 ng/L, as shown in Figure 2. The lowest level of BPA was observed upstream (SP5), while the highest was observed in the downstream zone (SP17). According to the Pollution Control Department’s water-source classification (Table S1, which categorizes water types based on standard physicochemical parameters such as DO and biochemical oxygen demand (BOD)), BPA concentration exhibited a clear increasing trend from type 2 to type 4 water sources. The upstream zone, classified as type 2, showed low BPA levels (32.29 ± 12.13 ng/L), reflecting minimal human impact and superior water quality. In contrast, the middle stream (type 3) showed moderate BPA levels (78.94 ± 11.51 ng/L), likely influenced by agricultural runoff and urban wastewater. The downstream zone (type 4) exhibited the highest concentrations (86.98 ± 24.33 ng/L), potentially due to industrial discharge, municipal wastewater, and reduced dilution capacity within the estuary.

**Figure 3:**
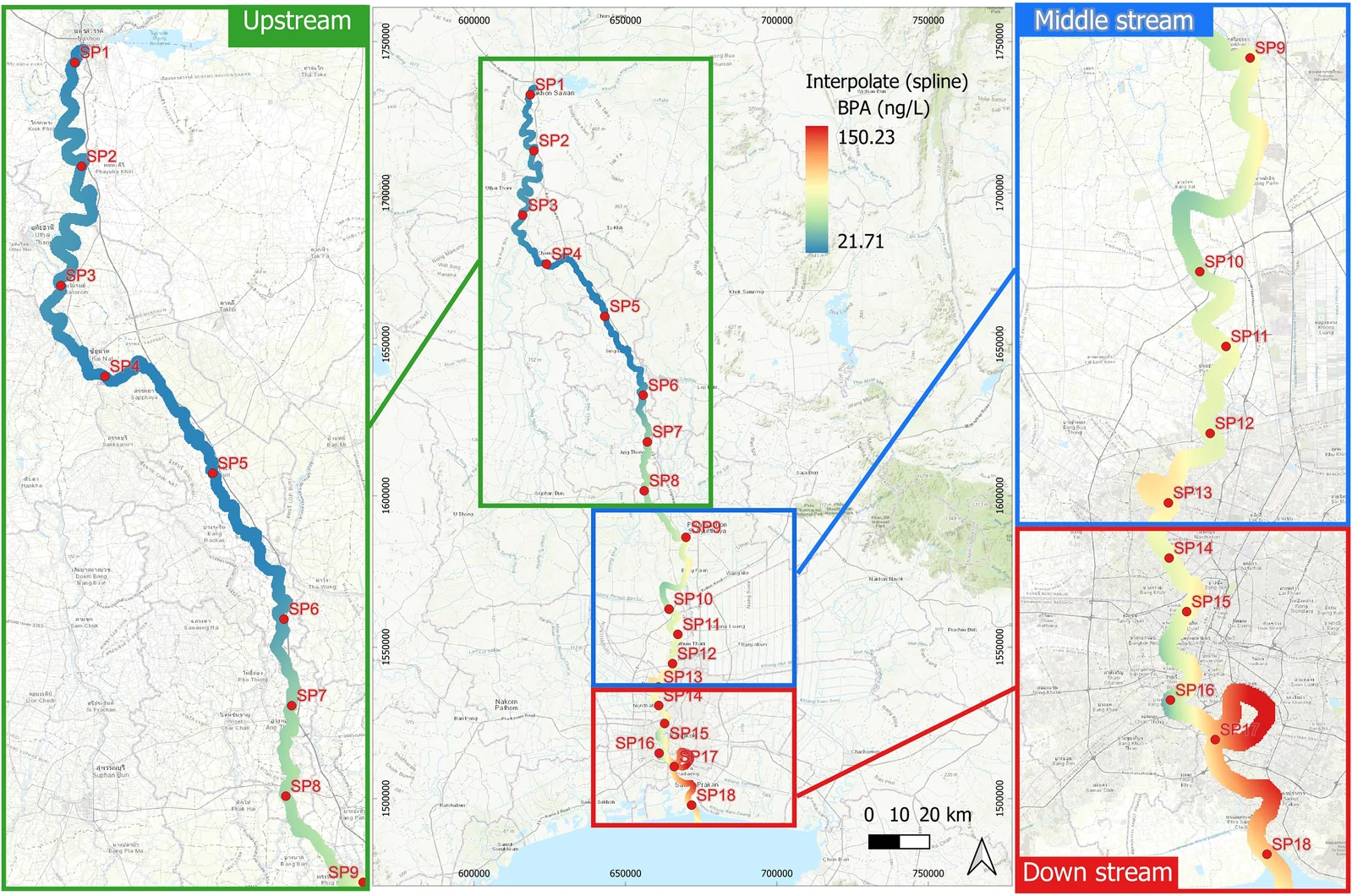
Spatial distribution of BPA concentrations in the upstream, middle stream, and downstream zones of the Chao Phraya River Scale bars, 20 km.

Spatial distribution analysis using spline interpolation (Figure 3) further illustrates the clear longitudinal gradient of BPA, with concentrations progressively increasing toward the downstream reaches. In this study, the interpolated surface was used primarily as a visualization tool to highlight the overarching spatial trend across the river basin, an approach previously applied in similar aquatic monitoring studies.^34^^,^^35^ However, we acknowledge that interpolation assumes spatial continuity and may not fully capture abrupt variations caused by localized point-source discharges or hydrodynamic processes. Therefore, Figure 3 should be interpreted as a representation of the basin-wide distribution pattern, which is consistent with the intensified anthropogenic pressures and declining DO observed downstream, rather than a tool for precise concentration estimates at unsampled locations.

While BPA is not yet a regulated parameter under the Thailand Surface Water Quality Standards (Table S2), its distribution effectively reflects the water quality degradation identified by the existing classification system. In this study, BPA levels remained well below the European Union’s drinking water parametric value of 2,500 ng/L.^36^ However, the concentrations at several sites exceeded the proposed European Union environmental quality standard (EQS) for surface water (0.17 ng/L),^37^ suggesting a potential long-term risk to the aquatic ecosystem of the Chao Phraya River.

Compared with other Asian rivers (Table 2), BPA levels in the Chao Phraya River were within reported ranges, indicating moderate contamination, as they fall intermediately between heavily polluted catchments and strictly managed waterways. Higher concentrations have been reported in the Nan River, Thailand (up to 1,554 ng/L),^20^ which is one of the four primary tributaries forming the Chao Phraya River, and the Liuxi River, China (up to 7,480 ng/L).^38^ These elevated levels are primarily attributed to untreated industrial and domestic wastewater discharges. Similar moderate BPA concentrations have been observed in the Selangor River, Malaysia (25.4 to 358 ng/L), reflecting high urban intensity.^40^ In contrast, rivers in Vietnam and other Malaysian reaches, such as the Bentong and Langat rivers, show significantly lower BPA levels (<10 ng/L) due to effective centralized wastewater treatment and limited industrial activity.^29^^,^^39^^,^^42^ Beyond infrastructure and anthropogenic inputs, this inter-study variability is heavily influenced by hydrological conditions and analytical methodologies. Hydrologically, heavy monsoon precipitation can significantly dilute BPA concentrations, as demonstrated in the Qinhuai River Basin (4.63–394 ng/L) where levels were notably higher before the flood period.^23^ Additionally, river flow dynamics between high- and low-flow periods can cause site-specific fluctuations.^15^ Analytically, disparities in detection methods contribute to reported differences. As detailed in Table 2, studies utilizing GC-MS^20^^,^^38^ require derivatization and possess different sensitivities compared to those employing liquid chromatography-tandem mass spectrometry (LC-MS/MS) or UPLC-QTOF-MS.^40^^,^^41^ Therefore, varying limits of detection, seasonal flow fluctuations, and regional waste management practices must be evaluated collectively when interpreting concentration ranges across different geographical regions.

**Table 2:** Comparison of BPA concentrations in surface waters from selected rivers across Asia

Country	Sampling location (number of samples)	Sampling period	Analytical method	Concentration (ng/L)	Reference
Thailand	Chao Phraya River (*n* = 18)	Oct 2023	SPE + UPLC-QTOF	22.11–113.07	this study
Thailand	Nan River (*n* = 12)	Aug 2014	SPE + GC-MS	34.97–1554.14	Deemoon et al.^20^
China	Qinhuai River Basin (*n* = 32)	Jun 2023	SPE + HPLC–MS/MS	4.63–394.00	Zhanget al.^23^
China	Liuxi River (*n* = 28)	Dec 2016	SPE + HPLC	75.60–7480.00	Huang et al.^38^
China	Yongding River (*n* = 27)	Jan 2022	SPE + UPLC	ND – 5.30	Honget al.^25^
China	Beiyun River (*n* = 8)	Jan 2022	SPE + UPLC	ND – 1.30	Honget al.^25^
Malaysia	Bentong River (*n* = 7)	Mar, Nov 2019	SPE + LC-MS/MS	1.13–5.52	Shehabet al.^29^
Malaysia	Langat River (*n* = 5)	Oct 2018	SPE + LC-MS/MS	1.18–8.24	Weeet al.^39^
Malaysia	Selangor River Basin (*n* = 25)	–	SPE + LC-MS/MS	25.43–358.05	Zaki et al.^40^
Philippines	Marikina River (*n* = 3)	Jan 2023	SPE + LC-MS/MS	28.52–54.26	Katrina et al.^41^
Philippines	Pasig River (*n* = 3)	Jan 2023	SPE + LC-MS/MS	30.47–54.23	Katrina et al.^41^
Philippines	Angat River (*n* = 4)	Mar 2023	SPE + LC-MS/MS	4.57–19.84	Katrina et al.^41^
Philippines	Pampanga River (*n* = 5)	Mar 2023	SPE + LC-MS/MS	ND – 2.87	Katrina et al.^41^
Taiwan	Kao-Pin River (*n* = 120)	Jul 2004–Dec 2005	SPE + HPLC	ND – 4.23	Chen et al.^15^
Vietnam	Rivers in Bac Ninh (*n* = 21)	Jun and Jul 2021	LLE + LC-MS/MS	1.00–30.20	Nguyen et al.^42^

### Multivariate analysis of BPA influencing factors

**Figure 4:**
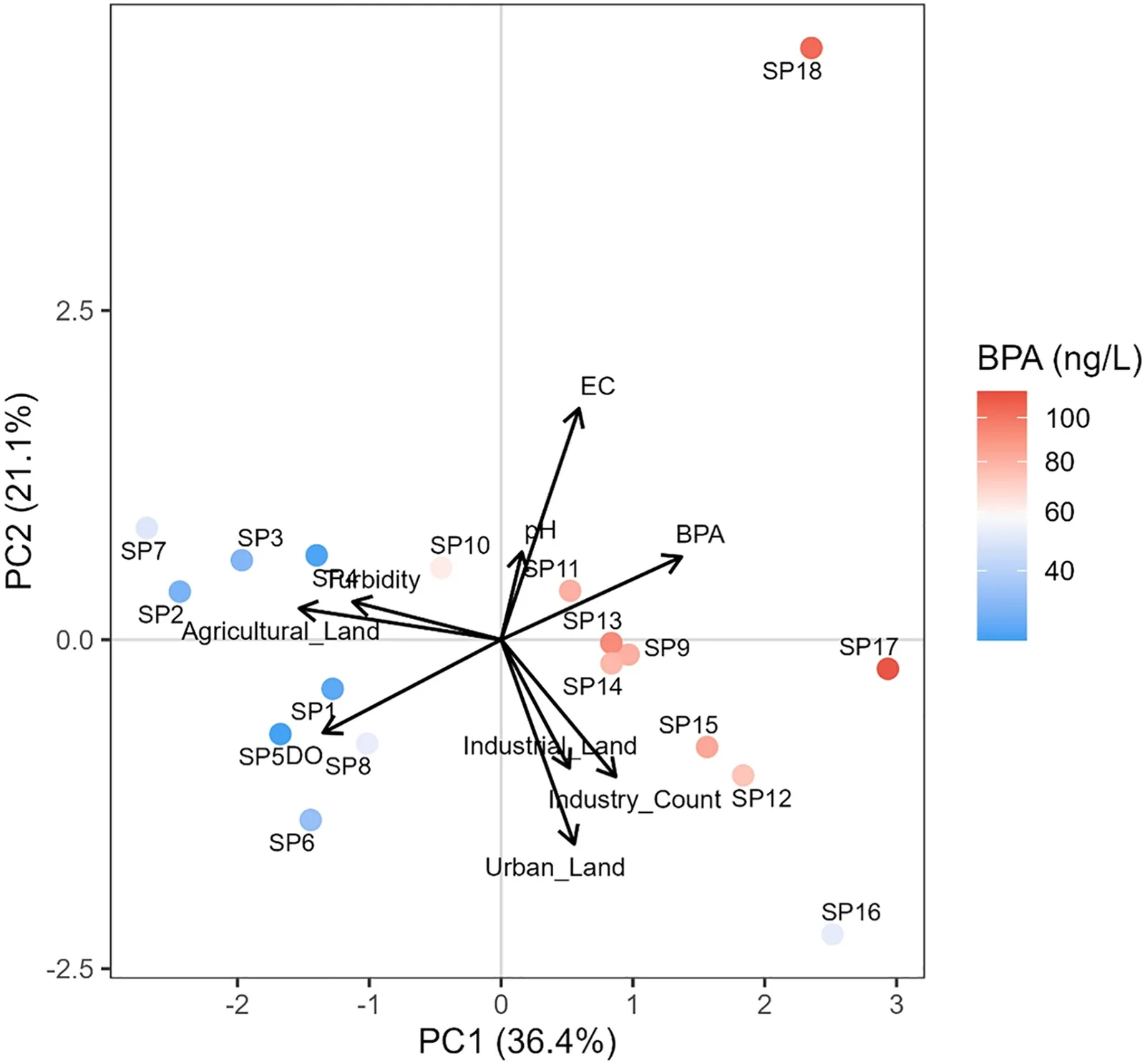
PCA biplot of BPA and environmental variables Points represent sampling sites colored by BPA concentration; arrows indicate variable loadings for PC1 and PC2.

PCA identified two dominant axes explaining the spatial variability of BPA (Figure 4; Table S6), with detailed scree plots and variable contributions provided in the supplemental information (Figures S1 and S2). PC1, which accounted for 36.4% of the total variance, served as the primary indicator for BPA contamination, as evidenced by the high positive loading for BPA (+0.456). This axis represented an urban and industrial gradient, showing strong positive associations with industrial land-use proportions, urban land-use proportions, and the total number of industrial facilities. Consequently, sites with intensive industrial and urban activity, such as SP11, SP13, SP14, and SP17, aligned along this axis and exhibited elevated BPA levels. This finding aligns with previous studies indicating that BPA concentrations peak near industrial clusters and wastewater-impacted reaches.^43^^,^^44^ PC2 explained 21.1% of the variance and was characterized by a positive loading for both BPA (+0.210) and pH (+0.222), while showing a strong negative loading for DO (–0.236). Although BPA was more strongly associated with PC1, its relationship with PC2 highlights that higher concentrations frequently coincide with oxygen depletion. Sites such as SP17 and SP18 were notably associated with low DO, supporting the observation that BPA tends to persist under oxygen-poor conditions due to reduced aerobic degradation.^43^ In contrast, agricultural sites (SP2–SP7) clustered in the quadrant corresponding to higher DO and lower BPA, a pattern typical of catchments dominated by non-industrial land use.^45^

The optimal MLR model (Table 3; Figure S3), selected by stepwise Akaike’s information criterion (AIC), retained pH, turbidity, DO, industrial land-use proportion, and industrial facility count as significant predictors and explained 85.9% of BPA variability (adjusted R^2^ = 0.801, *p* < 0.001). DO was the strongest predictor (β = −22.90, *p* < 0.001), indicating that each 1 mg/L increase in DO was associated with a substantial reduction in BPA concentration. This finding supports earlier reports that low-oxygen conditions are associated with higher persistence of BPA due to slower microbial degradation.^43^ Furthermore, this strong inverse relationship highlights the practical implications of the regulatory water classifications (Table S2). Different water types are governed by distinct physicochemical thresholds. For instance, the acceptable DO limit for type 4 industrial waters (≥2.0 mg/L) is significantly lower than that for type 2 conservation waters (≥6.0 mg/L). Consequently, the degraded water quality permitted in lower-tier classes inherently provides an environment that favors BPA persistence and accumulation. Regarding anthropogenic predictors, the proportion of industrial land use showed a significant positive association with BPA (+238.88, *p* = 0.023), confirming that large-scale industrial zones are major contributors.^23^^,^^44^^,^^46^ Conversely, industrial facility count exhibited a negative coefficient (–0.745, *p* = 0.0074). When the total industrial area constant is held, a higher facility count typically reflects a dense cluster of small-scale or light industries that do not emit BPA. This indicates that the scale of heavy manufacturing, such as plastics or resin production, is the primary driver of BPA contamination rather than the sheer number of small enterprises.^23^^,^^44^^,^^46^ Importantly, model diagnostics confirmed that this inverse relationship is not a statistical artifact of multicollinearity, as all variance inflation factor (VIF) values remained well below 1.5. Furthermore, the residuals were normally distributed (Shapiro-Wilk *p* = 0.381), validating the robustness of the current model specification according to standard econometric and multivariate analysis criteria.^47^^,^^48^

**Table 3:** Regression coefficients from the optimal multiple linear regression model predicting BPA concentrations (ng/L)

Predictor	Estimate	SE	*t* value	*p* value
(Intercept)	403.19	131.37	3.07	**0.0097**
pH	−33.57	18.22	−1.84	0.0902
Turbidity	−0.11	0.07	−1.67	0.1201
**DO**	**−22.90**	**3.17**	**−7.24**	**1.03**×**10**^**-5**^
**Industrial land-use proportion**	**238.88**	**91.67**	**2.61**	**0.0230**
**Industrial facility counts**	**−0.75**	**0.23**	**−3.22**	**0.0074**

Significant predictors (*p* < 0.05) are bolded. Model performance: R^2^ = 0.86; adjusted R^2^ = 0.80; *F* test *p* value < 0.001; VIF range = 1.1–1.5; Shapiro-Wilk *p* = 0.38.

Overall, the combined PCA and MLR results demonstrate that industrial activity and reduced water quality, particularly low DO, are the primary drivers of BPA contamination in the study area. This spatial trend in the Chao Phraya River closely aligns with the pattern reported in the Qinhuai River, China, where PCA-MLR analyses similarly identified wastewater discharge and urban runoff as the predominant sources of contamination.^23^ Furthermore, agricultural regions consistently exhibited lower BPA concentrations, a trend that aligns with patterns observed in other river basins worldwide where industrial point sources dominate bisphenol pollution.^23^^,^^43^^,^^45^

Given that DO is the primary predictor of BPA concentration, management interventions should focus on mitigating oxygen depletion to enhance the natural attenuation of bisphenols. Implementing stricter controls on organic load discharge from municipal and industrial sources is essential to reduce biochemical oxygen demand. Additionally, localized interventions such as artificial aeration in stagnant or heavily impacted downstream reaches could facilitate aerobic microbial degradation, thereby reducing BPA persistence.^23^^,^^43^ Such integrated water-quality management, combined with upgraded wastewater treatment infrastructure, would be critical for mitigating EDC pollution in the Chao Phraya River.

### Ecological risk assessment

**Figure 5:**
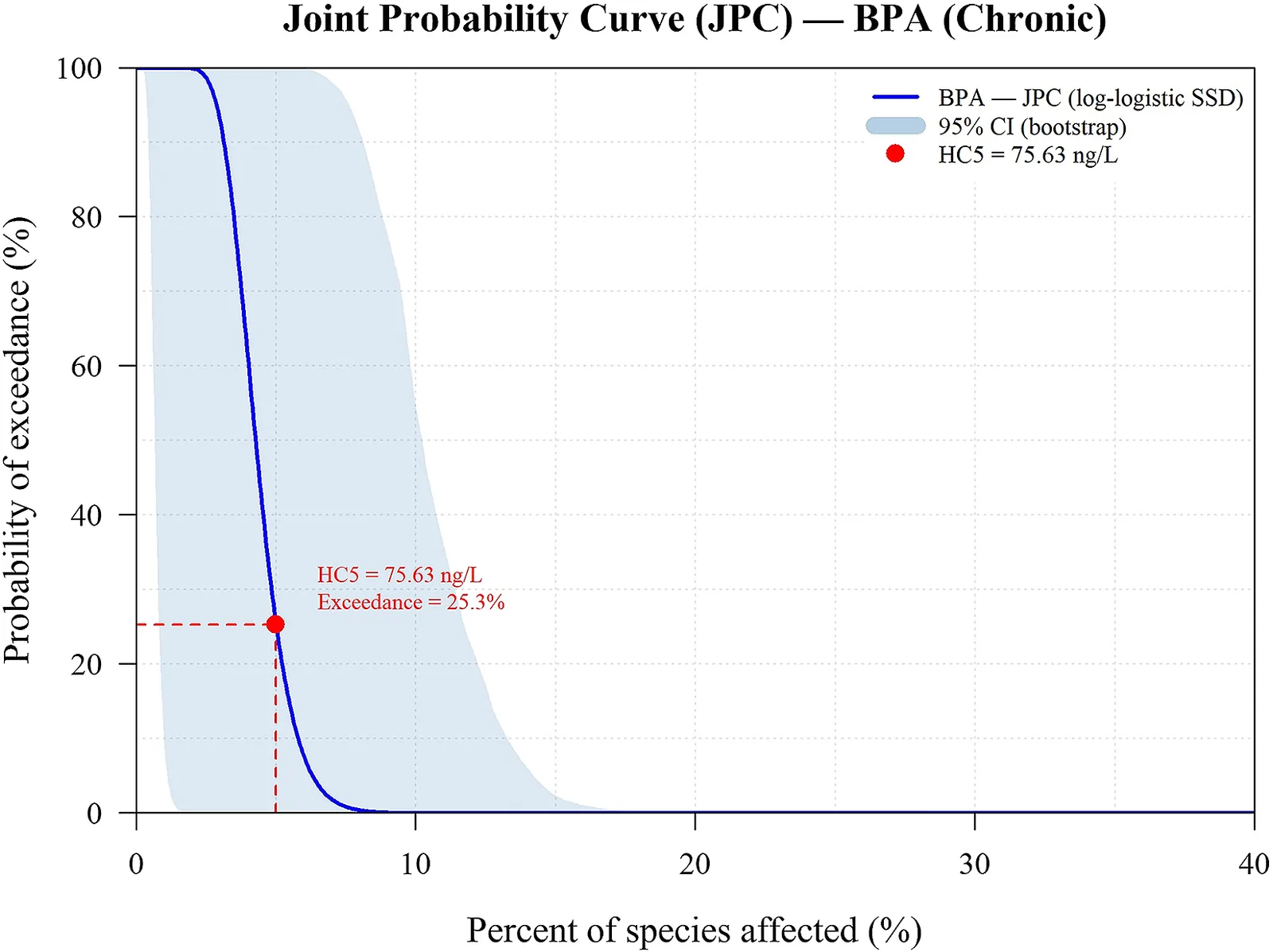
Joint probability curve for chronic ecological risk of BPA in the Chao Phraya River The shaded area represents the 95% bootstrap confidence interval.

The SSD curves for BPA, constructed using toxicity data from the US EPA ECOTOX database (Tables S3 and S4), are presented in Figure S4. The derived hazardous concentrations for 5% of species (HC5) were 1,08,451.65 ng/L for acute toxicity and 75.63 ng/L for chronic toxicity, as shown in Figure 5. Based on these probabilistic thresholds, the average acute RQ along the river was 2.79 × 10^-3^ (ranging from 0.0010 to 0.0052), indicating negligible acute risk across all sampling sites. In contrast, chronic RQ values exhibited a widespread high-risk scenario (RQ > 1) at every sampling location, with a river-wide average of 4.00 (ranging from 1.43 to 7.48), as summarized in Table 4. A distinct longitudinal risk gradient was observed across the defined water zones. In the upstream zone, the average chronic RQ was 2.13, representing high chronic risk even in areas with lower anthropogenic impact. This risk further intensified in the middle stream, where the average RQ rose to 5.22, and reached its peak in the downstream zone with an average chronic RQ of 5.75. These results demonstrate that while acute lethality is unlikely, current BPA concentrations in the Chao Phraya River pose a widespread high chronic risk, potentially affecting at least 5% of aquatic species.

**Table 4:** Acute and chronic risk quotients of BPA for aquatic communities at different sampling points and water zones in the Chao Phraya River

Sampling point		Acute RQ	Risk classification	Chronic RQ	Risk classification
Upstream	SP 1	1.10 × 10^-3^	no risk	1.57	high risk
–	SP 2	1.28 × 10^-3^	no risk	1.83	high risk
–	SP 3	1.33 × 10^-3^	no risk	1.91	high risk
–	SP 4	1.02 × 10^-3^	no risk	1.46	high risk
–	SP 5	9.96 × 10^-4^	no risk	1.43	high risk
–	SP 6	1.48 × 10^-3^	no risk	2.13	high risk
–	SP 7	2.29 × 10^-3^	no risk	3.28	high risk
–	SP 8	2.42 × 10^-3^	no risk	3.46	high risk
**Average upstream**		**1.49 × 10**^**-3**^	no risk	**2.13**	high risk
Middle stream	SP 9	3.77 × 10^-3^	no risk	5.41	high risk
–	SP 10	2.88 × 10^-3^	no risk	4.13	high risk
–	SP 11	3.76 × 10^-3^	no risk	5.40	high risk
–	SP 12	3.45 × 10^-3^	no risk	4.95	high risk
–	SP 13	4.33 × 10^-3^	no risk	6.21	high risk
**Average middle stream**		**3.64 × 10**^**-3**^	no risk	**5.22**	high risk
Downstream	SP 14	3.63 × 10^-3^	no risk	5.20	high risk
–	SP 15	3.90 × 10^-3^	no risk	5.59	high risk
–	SP 16	2.39 × 10^-3^	no risk	3.43	high risk
–	SP 17	5.21 × 10^-3^	no risk	7.48	high risk
–	SP 18	4.91 × 10^-3^	no risk	7.05	high risk
**Average downstream**		**4.01 × 10**^**-3**^	no risk	**5.75**	high risk
**Average along river**		**2.79 × 10**^**-3**^	no risk	**4.00**	high risk

The bold values represent the average values calculated for each sampling zone (upstream, middle stream, and downstream), rather than statistical significance.

The JPC analysis further confirmed this ecological threat, showing a significant overlap between exposure concentrations and species sensitivity. Approximately 25.3% of the monitored concentrations exceeded the chronic HC5 (75.63 ng/L), suggesting a high likelihood of adverse ecological effects under typical environmental conditions. The estimated overall risk probability (ORP) was 30.2% (25%–35%), which falls within the high ecological risk category, demonstrating that chronic BPA impacts persist under typical environmental conditions.

The chronic ecological risks observed in the Chao Phraya River align with patterns reported elsewhere. Zhang et al. found that BPA frequently presents medium to high chronic risk for invertebrates and fish in urbanized rivers,^23^ while Santos et al. also identified these groups as the most sensitive across global water bodies.^49^ Our findings are further supported by Tišler et al., who reported strong chronic effects on Daphnia magna and zebrafish at low levels,^50^ and by Bhandari et al., who documented reproductive and developmental impacts in fish at environmentally relevant concentrations.^51^ Collectively, the evidence indicates that chronic BPA exposure poses a substantial ecological threat in the Chao Phraya River, driven by sustained pollutant inputs and decreasing dilution capacity toward the estuary.

### Human health risk assessment

The non-carcinogenic risk of BPA exposure, expressed as hazard quotient (HQ), was well below the safety threshold (HQ = 1) across all sampling sites and population groups (Table S7), indicating negligible health risk for both adults and children. These findings suggest that using raw water from the Chao Phraya River as a drinking water supply source is unlikely to pose significant health risks related to BPA exposure.

Furthermore, previous studies have demonstrated that conventional water treatment processes can remove approximately 60%–90% of BPA from raw water.^13^^,^^14^^,^^52^^,^^53^ Therefore, the actual exposure levels after drinking water treatment are expected to be even lower than those estimated in this study, further supporting the conclusion of negligible health risk.

### Limitations of the study

This study provides a basin-scale assessment of BPA occurrence, controlling factors, and associated ecological and human health risks in the Chao Phraya River. However, the findings represent a single sampling campaign conducted during the wet season and therefore may not fully capture seasonal variations associated with hydrological conditions. In addition, the identified relationships between BPA and environmental variables were based on statistical associations rather than direct mechanistic investigations. Future studies incorporating multi-season monitoring, hydrological modeling, and source-specific analyses would further improve the understanding of BPA transport, fate, and long-term ecological impacts in tropical river systems.

## Resource availability

### Lead contact

Requests for further information and resources should be directed to and will be fulfilled by the lead contact, Kraichat Tantrakarnapa (kraichat.tan@mahidol.ac.th).

### Materials availability

This study did not generate new unique reagents.

### Data and code availability

• Data reported in this paper will be shared by the lead contact upon request.
• This paper does not report original code.
• Any additional information required to reanalyze the data reported in this paper is available from the lead contact upon request.

## Acknowledgments

This research project is supported by the Royal Golden Jubilee PhD, 10.13039/501100004704National Research Council of Thailand (NRCT): NRCT5-RGJ63012-137 to S.N. We also thank the Department of Social and Environmental Medicine; the Environment, Health & Social Impact Unit, Faculty of Tropical Medicine, Mahidol University, Thailand; Research Institute for Health Sciences, Chiang Mai University; and the Rollins School of Public Health, Emory University for providing support, equipment, and facility.

## Author contributions

Conceptualization, S.N., W.K., T.C.B., K.S., Y.L., S.W., S.H., P.P., K.C., D.I., and K.T.; methodology, S.N., W.K., D.I., and K.T.; investigation, S.N., P.P., K.C., D.I., and K.T.; formal analysis, S.N., R.M., N.K., T.C.B., K.S., and W.P.; data curation, S.N., R.M., and N.K.; visualization, W.P.; writing – original draft, S.N.; writing – review & editing, S.N., W.K., T.C.B., Y.L., S.W., S.H., W.P., P.P., K.C., D.I., and K.T.; supervision, Y.L., S.W., S.H., P.P., K.C., D.I., and K.T.; validation, S.N. and T.C.B.; funding acquisition, S.N. and D.I.

## Declaration of interests

The authors declare no competing interests.

## Declaration of generative AI and AI-assisted technologies in the writing process

During the preparation of this work, the authors used ChatGPT in order to improve the writing style. After using this tool/service, the authors reviewed and edited the content as needed and take full responsibility for the content of the publication.

## STAR★Methods

### Key resources table

**Table:** 

REAGENT or RESOURCE	SOURCE	IDENTIFIER
**Chemicals, peptides, and recombinant proteins**		

Bisphenol A (BPA) analytical standard	Dr. Ehrenstorfer, Augsburg, Germany	Cat#C10655500
BPA-propane-D6 internal standard	Dr. Ehrenstorfer, Augsburg, Germany	Cat#DLM-2775–0.1
Phenomenex Strata-X SPE cartridge (33 μm, 500 mg/6 mL)	Phenomenex	Cat#8B-S100-HCH
Kinetex EVO C18 100 Å column (150 × 2.1 mm, 1.7 μm)	Phenomenex	Cat#00F-4726-AN
PTFE syringe filter (0.2 μm, 4 mm, VertiPure)	VertiPure	Cat#0200–2101, LOT#3891

**Software and algorithms**		

R version 4.3.3	R Foundation for Statistical Computing	https://cran.r-project.org
RStudio 2025.09.2 + 418	Posit Software	https://posit.co
QGIS version 3.40	QGIS Development Team	https://qgis.org
ggplot2	R package	https://ggplot2.tidyverse.org
ggrepel	R package	https://cran.r-project.org/package=ggrepel
factoextra	R package	https://cran.r-project.org/package=factoextra

**Other**		

HQ40d portable multiparameter meter	Hach Company	Model HQ40d
Agilent 6546 LC/Q-TOF system	Agilent Technologies	Model 6546

### Method details

#### Description of the study area

Chao Phraya River basin is situated in central Thailand and spans an area of approximately 20,523.42 square kilometers. The river originates at the confluence of the Ping and Nan Rivers in Nakhon Sawan Province and flows southward for 372 kilometers.^30^ The Pollution Control Department has classified the Chao Phraya River into three zones, namely upstream, middle stream, and downstream, to define the types of surface water sources and their intended uses,^54^^,^^55^ as presented in Table S1 and surface water quality standard in Table S2.

#### Water sample collection, extraction and analysis

At each site, 2L. of surface water was collected using a vertical grab water sampler (1L. capacity), requiring two consecutive grab collections that were combined to obtain one representative sample per site. Details of the sampling locations are provided in Figure 1; Table 1. To ensure sample integrity, all samples were stored in pre-cleaned DURAN bottles rinsed with methanol, placed in iceboxes, and maintained at approximately 4 °C during transport to the laboratory. Key physicochemical parameters, including temperature, pH, dissolved oxygen (DO), electrical conductivity (EC), and turbidity, were measured *in situ* using a portable multiparameter meter (HQ40d, Hach Company, USA).

Sample extraction and analysis followed a modified protocol based on the U.S. Environmental Protection Agency^56^ and Wee et al.^39^ 2L. of water samples were filtered through 0.2 μm glass microfiber filters, and the pH was adjusted to 2.0 ± 0.5 with 1 M HCl. Before extraction, 100 μL of internal standard and 1 g of disodium ethylenediaminetetraacetic acid (Na_2_EDTA) were added and equilibrated for 2 h. Solid-phase extraction (SPE) was performed using Phenomenex Strata-X polymeric reversed-phase cartridges (33 μm, 500 mg/6 mL). Cartridges were conditioned with 20 mL of methanol, followed by 6 mL of ultrapure water and 6 mL of acidified ultrapure water (pH 2.0 ± 0.5), ensuring the sorbent remained continuously wet between steps. Prepared samples were loaded at a flow rate of 5–10 mL/min using a vacuum pump, rinsed with 10 mL of ultrapure water, and dried under vacuum for 15 min. Analytes were eluted sequentially with 10 mL of methanol, 5 mL of a 1:1 (v/v) methanol–acetone mixture, and 5 mL of dichloromethane. The eluates were combined and evaporated to dryness under nitrogen, reconstituted in 1 mL of methanol, filtered through 0.2 μm PTFE syringe filters (4 mm, VertiPure), and stored in 2 mL amber glass vials at −20 °C prior to instrumental analysis.

BPA was quantified using an Agilent 6546 LC/Q-TOF system integrating ultra-performance liquid chromatography with a quadrupole time-of-flight mass spectrometer (UPLC-QTOF-MS) operated in negative electrospray ionization mode. The mobile phase consisted of 0.2% ammonium hydroxide (NH_4_OH) in water (A) and methanol (B). Chromatographic separation was achieved on a Kinetex EVO C18 100A column (150 × 2.1 mm, 1.7 μm) using a gradient starting at 60% B, decreasing to 50% at 5 min, and increasing to 100% B at 17 min. The injection volume was 1 μL, the column temperature was maintained at 35 °C, and the flow rate was 0.3 mL/min. The drying gas flow rate was 11 L/min at 275 °C.

#### Quality assurance and control (QA/QC)

Quality assurance and control procedures were implemented throughout the analytical process. Method blanks prepared with deionized water were extracted alongside samples to monitor background contamination. Isotopically labeled internal standards (BPA-propane-D6) and target standards were obtained from Dr. Ehrenstorfer (Augsburg, Germany). Real-time mass calibration was maintained by continuous infusion of reference ions (positive mode: 121.0509, 922.0098; negative mode: 68.9957, 112.9855, and 301.9981). Instrument precision and accuracy were confirmed through calibration curves (R^2^ = 0.9994) and recovery tests, with BPA recovery of 120.1% and %CV of 2.74. According to US EPA criteria, acceptable recoveries for water analysis range between 70% and 130%.^56^ The relatively high recovery (120.1%) suggests potential matrix-induced signal enhancement, likely due to complex organic constituents in river water, potentially leading to a slight overestimation of BPA concentrations. Future studies could apply isotope dilution approaches to further improve analytical accuracy and minimize matrix effects.

#### Statistical analysis

PCA was used to explore multivariate relationships between BPA concentrations and environmental and land-use variables. The variables included BPA, pH, turbidity, DO, and EC, as well as agricultural, urban, and industrial land-use proportions obtained from the Land Development Department, and the number of industrial facilities obtained from the Department of Industrial Works. All variables were standardized (mean = 0, SD = 1), and PCA was performed using the prcomp() function in R with centering and scaling. Eigenvalues, variance explained, and variable loadings were examined to identify dominant environmental gradients, and biplots were produced using ggplot2, ggrepel, and factoextra.

Multiple Linear Regression (MLR) was conducted to identify predictors of BPA concentration. The initial model included all environmental and land-use variables, after which stepwise model selection based on Akaike’s Information Criterion (AIC) was applied to obtain the most parsimonious model.^57^ The final model retained pH, turbidity, DO, industrial land-use proportions, and number of industrial facilities as significant predictors. Model performance was evaluated using R^2^ and adjusted R^2^. Multicollinearity was assessed using the Variance Inflation Factor (VIF), with values < 5 considered acceptable. Model assumptions were checked using diagnostic plots and the Shapiro–Wilk test, and influential observations were identified using Cook’s distance (4/n threshold). All analyses were performed in R (version 4.3.3) using RStudio 2025.09.2+418.

#### Spatial distribution analysis

The spatial distribution of BPA concentrations was visualized using spline interpolation in QGIS (version 3.40). This method was selected over inverse distance weighted (IDW) interpolation as it produced a smoother and more interpretable representation of the longitudinal spatial gradient along the river system. It should be noted that the interpolation results were employed solely for geographical pattern visualization.

#### Ecological risk assessment

The ecological risk of BPA in the surface water of the Chao Phraya River was evaluated using a tiered probabilistic approach, that incorporated both the Risk Quotient (RQ) method based on Species Sensitivity Distributions (SSD) and the Joint Probability Curve (JPC) method.

##### Risk quotient (RQ)

The RQ was determined by comparing the measured environmental concentration (MEC) with the predicted no-effect concentration (PNEC). The RQ values were calculated as shown in Equation 1.

(Equation1) $$\;\text{RQ}=\frac{\text{MEC}}{\text{PNEC}}$$

where MEC represents the measured concentration of BPA (ng/L) in surface water, and PNEC is the predicted no-effect concentration which is derived from the SSD using the hazardous concentration affecting 5% of species (HC5), as shown in Equation 2.

(Equation 2) $$\text{PNEC}=\frac{\text{HC}5}{\text{AF}}$$

where HC5 represents the 5th percentile of the SSD, indicating the concentration at which 5% of aquatic species are potentially affected. An assessment factor (AF) of 5 was applied to the HC5 to account for residual uncertainties in species sensitivity and laboratory-to-field extrapolation. Toxicity data were compiled from the US EPA ECOTOX database including no-observed-effect concentrations (NOEC), 50% effect concentrations (EC50) and 50% lethal concentrations (LC50).^58^ The dataset included freshwater species native to Thailand, representing three trophic groups: algae, invertebrates (including crustaceans), and fish (Tables S3 and S4). Ecological risk levels were classified as negligible (RQ < 0.01), low risk (0.01 ≤ RQ < 0.1), medium risk (0.1 ≤ RQ < 1), and high risk (RQ ≥ 1).

##### Joint Probability Curve (JPC) and Overall Risk Probability (ORP)

To further characterize ecological risk, the JPC method was employed to integrate the exposure distribution of monitoring data with the chronic toxicity distribution of aquatic species.^59^^,^^60^ Both environmental concentrations and toxicity data were fitted to log-normal (exposure) and log-logistic (SSD) distributions. The Overall Risk Probability (ORP) was subsequently calculated as the area under the JPC, representing the cumulative probability of environmental concentrations exceeding toxicity thresholds.^61^ The ecological risk was categorized based on the ORP values as follows: negligible (ORP < 0.25%), low (0.25–2%), medium (2–10%), and high (ORP ≥ 10%).^62^

#### Human health risk assessment

The human health risk assessment for BPA in the Chao Phraya River was conducted following WHO guidelines.^63^ This assessment included exposure estimation and non-carcinogenic risk characterization. BPA was not evaluated for carcinogenic risk as it is not currently classified as a human carcinogen by major regulatory agencies. Under current water quality classifications, the upstream section of the Chao Phraya River is designated as Type 2, which is intended for general consumption after standard water treatment. Consequently, this study focused the health risk assessment exclusively on the upstream sampling locations to evaluate potential risks associated with its use as a raw water source for domestic consumption.

The risk characterization is based on the Estimated Daily Intake (EDI) and the Reference Dose (RfD), as shown in Equation 3. A Hazard Quotient (HQ) value greater than 1 indicates a potential health risk from exposure through drinking water and suggests the possibility of adverse health effects.

(Equation 3) $$\text{HQ}=\frac{\text{EDI}}{\text{RfD}}$$

The RfD for BPA is 0.05 mg/kg/day, based on reduced body weight gain in female rats and mice, which is considered a biomarker indicative of systemic toxicity and potential metabolic disruption.^64^ The EDI (mg/kg/day) is calculated using Equation 4.

(Equation 4) $$\text{EDI}=\frac{C\times \text{IR}\times \text{EF}\times \text{ED}}{\text{BW}\times \text{AT}}$$

Where C is the concentration of BPA in the upstream zone of the Chao Phraya River (mg/L), IR is the ingestion rate (L/day), EF is the exposure frequency (days/year), ED is the exposure duration (years), BW is the body wseight (kg), and AT is the averaging time (days). For this assessment, ED was assumed to be 30 years, representing a conservative default value for long-term residential exposure. AT was calculated as ED multiplied by 365 days. Detailed exposure factors and values used in this assessment are presented in Table S5.

## Supplemental information

Document S1. Figures S1–S4 and Tables S1–S3 and S5–S7

Table S4. Chronic toxicity data for bisphenol A used to derive the HC5 for ecological risk assessment, related to STAR Methods (ecological risk assessment) and Figure S4
