# Supplementary material for: Environmental drivers and ecological risks of bisphenol A occurrence in Chao Phraya River, Thailand
Source: iScience. 2026 Sep 7;29(9):117460. doi: 10.1016/j.isci.2026.117460 (PMC13578470; doi:10.1016/j.isci.2026.117460)
Supplement: Supplementary file 1 [file mmc1.pdf]

iScience, Volume *29*

Supplemental information

Environmental drivers and ecological risks of bisphenol A occurrence in Chao Phraya River, Thailand

Sarima Niampradit, Wissanupong Kliengchuay, Rachaneekorn Mingkhwan, Nuttapohn Kiangkoo, Tret C. Burdette, Kunrunya Sutan, Yanin Limpananont, Suwalee Worakhunpiset, Surat Hongsibsong, Walaiporn Phonphan, Parinya Panuwet, Kyungho Choi, Duangrat Inthorn, and Kraichat Tantrakarnapa

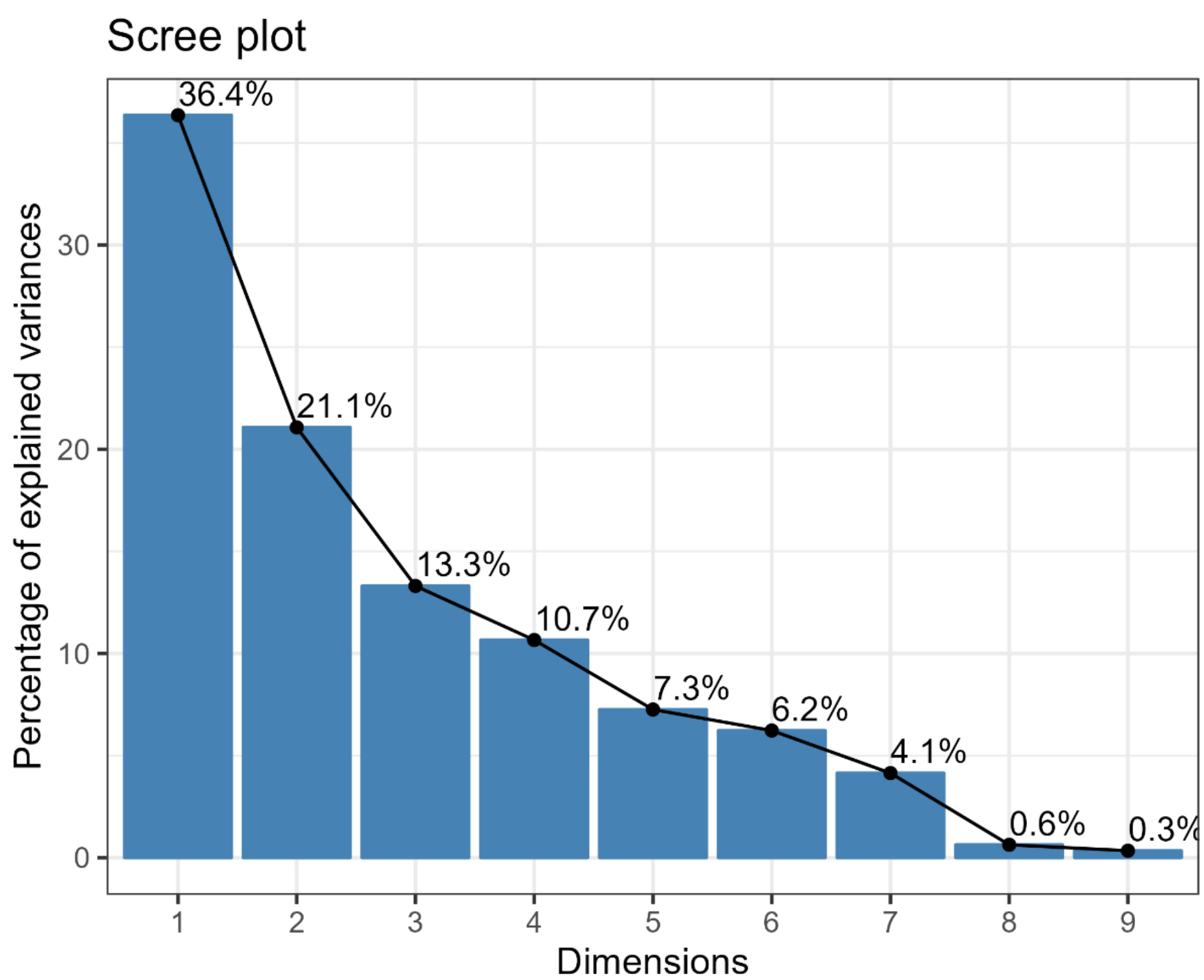

Figure S1. Scree plot showing the variance explained by principal components, Related to result in part 'Multivariate analysis of BPA influencing factors' and figure 4.

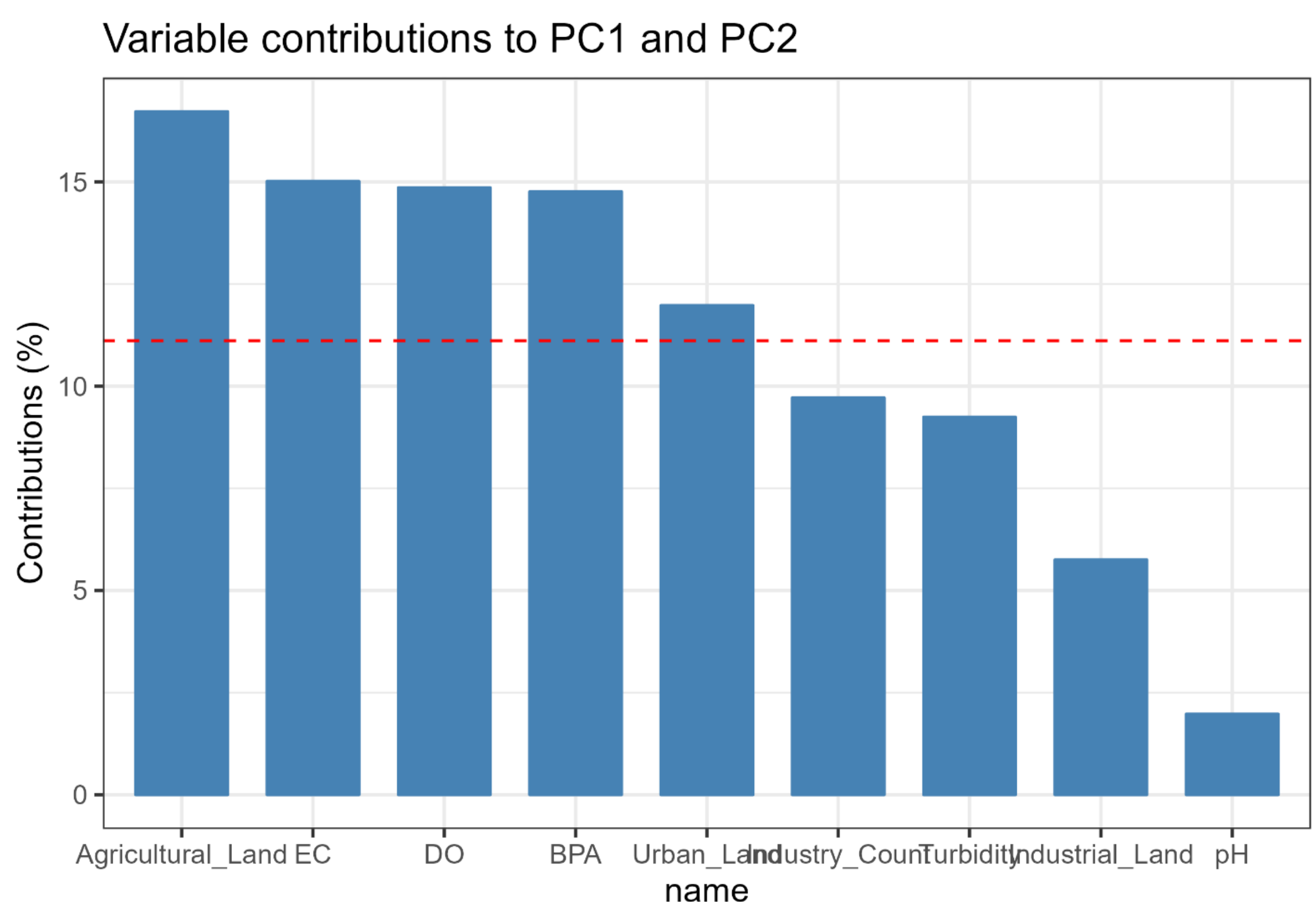

Figure S2. Contribution of environmental variables to PC1 and PC2, Related to result in part 'Multivariate analysis of BPA influencing factors' and figure 4

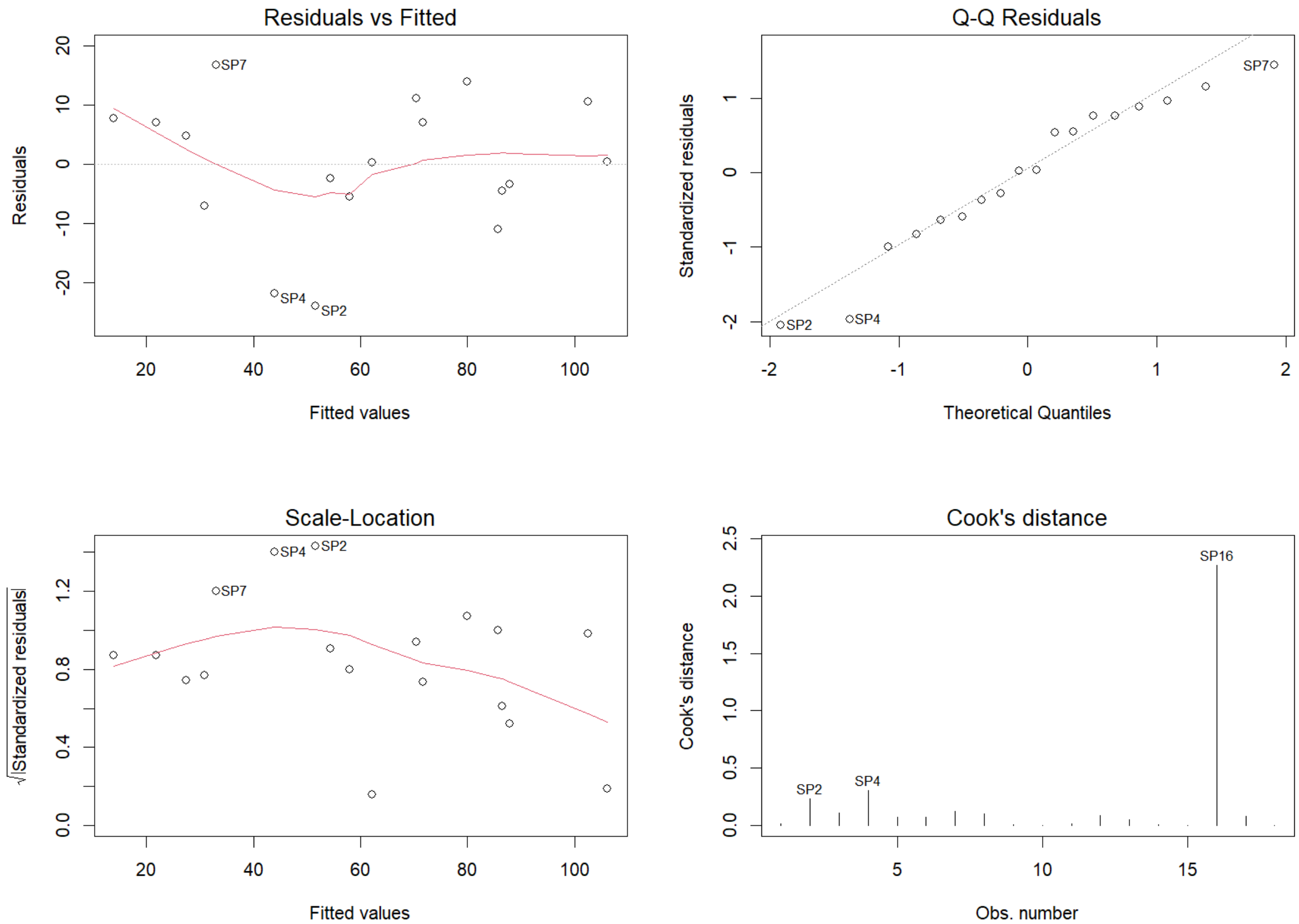

Figure S3. Diagnostic plots of the optimal multiple linear regression model. Residual vs. fitted, Q–Q plot, scale–location, and Cook's distance, Related to result in part 'Multivariate analysis of BPA influencing factors'

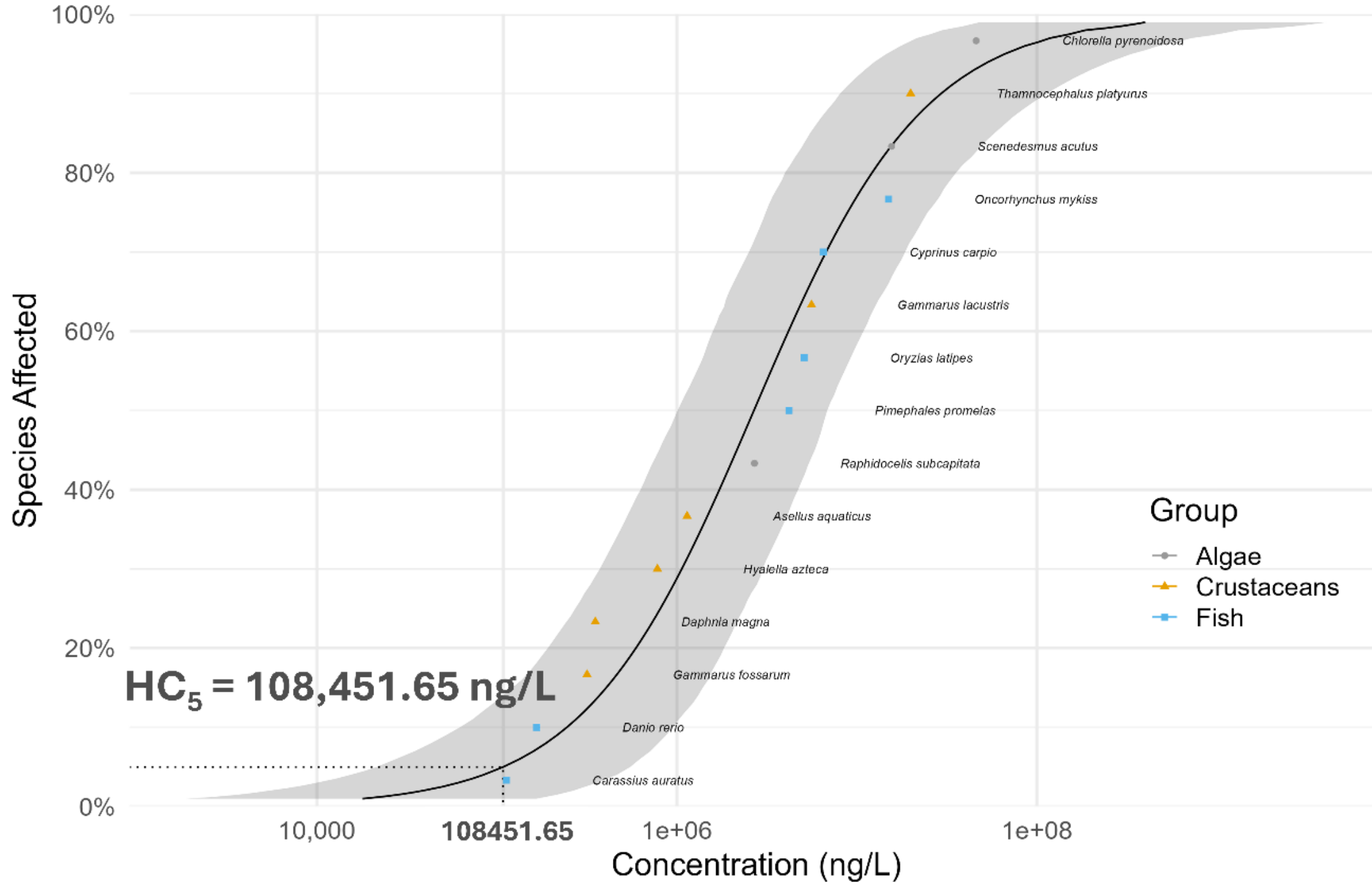

(a) Acute

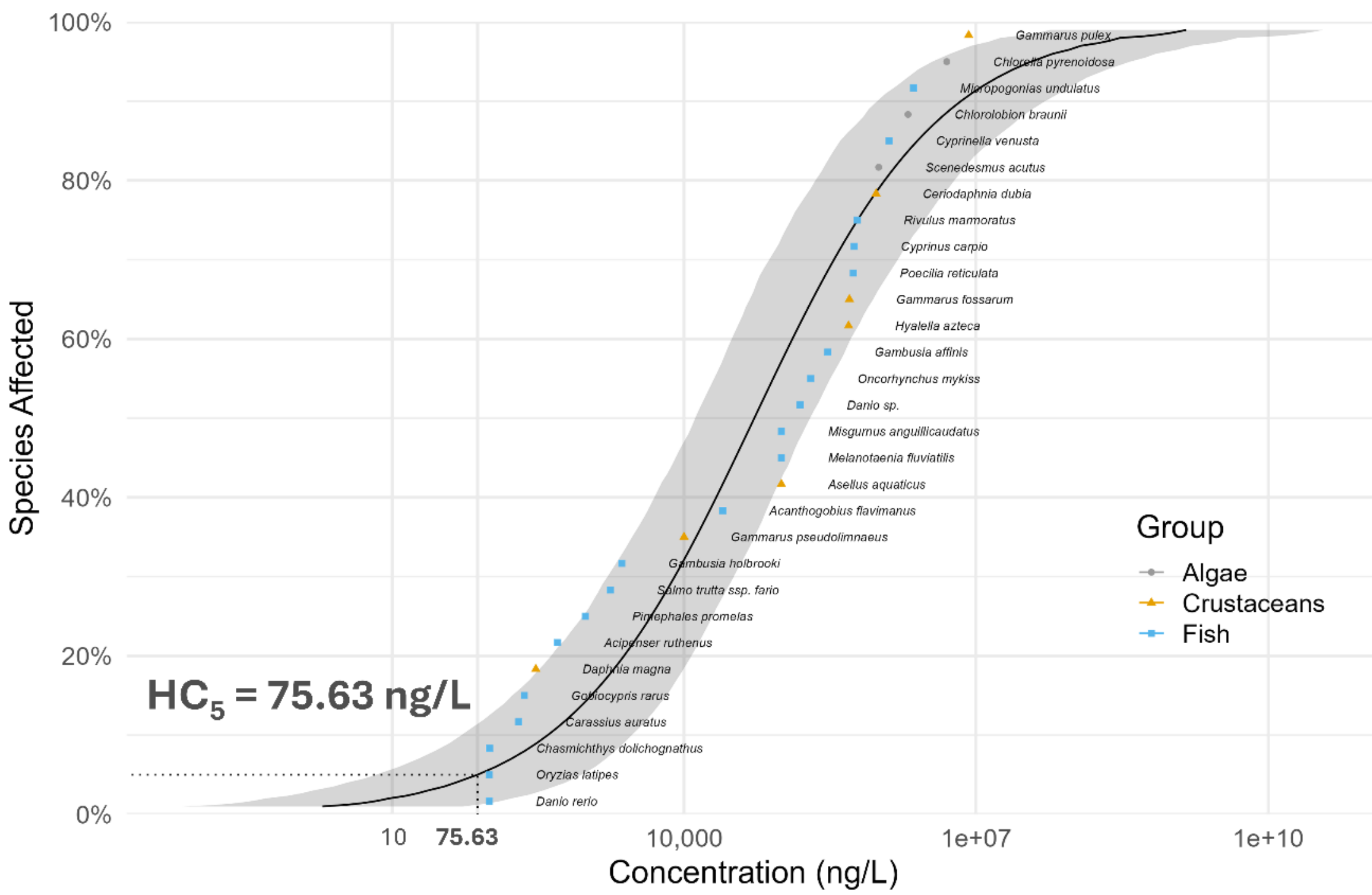

(b) Chronic

Figure S4 Species Sensitivity Distribution (SSD) curves for BPA based on (a) acute and (b) chronic toxicity data for the derivation of HC5 values. The shaded area represents the 95% bootstrap confidence interval. Related to Figure 5 and the Results section 'Ecological risk assessment'

Table S1. Classification of Water Quality Zones in the Chao Phraya River, Related to study area.

River zone	Location Description	Classification of Water Sources	Intended Use / Water Quality Objective
Upstream	From the pier in Phra Nakhon Si Ayutthaya District, Phra Nakhon Si Ayutthaya Province (river kilometer 68) upstream to the confluence with the Ping River in Nakhon Sawan Province (river kilometer 224)	Type 2 water source	For general consumption (after standard treatment), for aquatic life conservation, fisheries, and recreation
Middle stream	From the middle pier in Mueang District, Nonthaburi Province (river kilometer 26) upstream to the pier in Phra Nakhon Si Ayutthaya District, Phra Nakhon Si Ayutthaya Province (river kilometer 68)	Type 3 water source	For agriculture, livestock, and general industrial water supply after standard treatment.
Downstream	From Pak Nam Samut Prakan District, Samut Prakan Province (river kilometer 3) upstream to the middle pier in Mueang District, Nonthaburi Province (river kilometer 26)	Type 4 water source	For navigation and industrial use that does not require high-quality water.

Table S2 Thailand surface water quality standard, Related to result in part 'Occurrence of BPA in surface water of Chao Phraya River'.

Water parameter	Type 2	Type 3	Type 4
pH	5 – 9	5 – 9	5 – 9
DO (mg/L)	6.0	4.0	2.0
BOD (mg/L)	1.5	2.0	4.0
Total Coliform Bacteria (MPN/100 mL)	5,000	20,000	-
Fecal Coliform Bacteria (MPN/100 mL)	1,000	4,000	-

Table S3 Acute toxicity data for Bisphenol A, Related to result in part 'Ecological risk assessment' and Figure S4.

Organism	Species Name	Effect	Effect Measurement	Endpoint	Effect conc. (mg/L)
Algae	*Chlorella pyrenoidosa*	Population	Population growth rate	EC50	46.04
Algae	*Raphidocelis subcapitata*	Growth	Growth, general	EC50	2.7
Algae	*Raphidocelis subcapitata*	Population	Biomass	EC50	3.1
Algae	*Scenedesmus acutus*	Population	Population growth rate	EC50	15.59
Crustaceans	*Asellus aquaticus*	Feeding behavior	Food consumption	EC50	1.14
Crustaceans	*Asellus aquaticus*	Intoxication	Immobile	EC50	2.35
Crustaceans	*Asellus aquaticus*	Mortality	Mortality	LC50	9.5
Crustaceans	*Asellus aquaticus*	Physiology	Pigmentation	EC50	2.24
Crustaceans	*Daphnia magna*	Biochemistry	Calcein	EC50	2.56
Crustaceans	*Daphnia magna*	Biochemistry	Lipid	EC50	5.11
Crustaceans	*Daphnia magna*	Feeding behavior	Food consumption	EC50	22.69

Crustaceans	*Daphnia magna*	Intoxication	Immobile	EC50	0.35
Crustaceans	*Daphnia magna*	Mortality	Mortality	LC50	0.81
Crustaceans	*Daphnia magna*	Reproduction	Fecundity	EC50	7.3
Crustaceans	*Gammarus fossarum*	Intoxication	Immobile	EC50	1.9
Crustaceans	*Gammarus fossarum*	Reproduction	Progeny counts	EC50	0.32
Crustaceans	*Gammarus fossarum*	Reproduction	Reproducing organisms	EC50	1.01
Crustaceans	*Gammarus lacustris*	Intoxication	Immobile	EC50	5.6
Crustaceans	*Gammarus pulex*	Mortality	Mortality	LC50	1.49
Crustaceans	*Hyalella azteca*	Mortality	Survival	LC50	0.78
Crustaceans	*Thamnocephalus platyurus*	Mortality	Mortality	LC50	19.9
Fish	*Carassius auratus*	Biochemistry	Vitellogenin	EC50	0.11
Fish	*Cyprinus carpio*	Mortality	Mortality	LC50	6.53
Fish	*Danio rerio*	Development	Abnormal	EC50	4.56
Fish	*Danio rerio*	Genetics	Vitellogenin 1 mRNA	EC50	0.17
Fish	*Danio rerio*	Histology	Edema	EC50	13.9
Fish	*Danio rerio*	Morphology	Abnormal	EC50	11.3
Fish	*Danio rerio*	Mortality	Mortality	LC50	3.99
Fish	*Danio rerio*	Multiple	Multiple effects	EC50	10.55
Fish	*Danio rerio*	Physiology	Pigmentation	EC50	3.6
Fish	*Danio rerio*	Reproduction	Fertilization	EC50	1.40
Fish	*Danio rerio*	Reproduction	Hatch	EC50	4
Fish	*Oncorhynchus mykiss*	Cell(s)	Cytotoxicity	EC50	15
Fish	*Oryzias latipes*	Morphology	Abnormal	EC50	9.8
Fish	*Oryzias latipes*	Mortality	Mortality	LC50	5.1
Fish	*Pimephales promelas*	Biochemistry	Vitellogenin	EC50	0.16
Fish	*Pimephales promelas*	Mortality	Mortality	LC50	4.2

Table S5. Exposure factor definition and values used in human health risk assessment, Related to STAR methods (Human health risk assessment).

Parameter	Definition	Value	Unit	Reference
RfD	Reference dose	0.05	mg/kg/day	USEPA, 1993
ED	Exposure duration	30	Year	
AT	Average time	10,950	Day	
EF	Exposure frequency	365	Days/Year	
IR	Ingestion rate		L/Days	EFSA, 2010
	- Female adult	2.0		
	- Male adult	2.5		
	- Children	1.6		
BW	Body weight		kg	Kukusamude et al., 2021
	- Female adult	56.68		
	- Male adult	61.89		
	- Female children	19.73		
	- Male children	20.18		

Table S6. PCA loadings of environmental variables on PC1 and PC2, Related to result in part 'Multivariate Analysis of BPA Influencing Factors' and figure 4

Variable	PC1 (36.4%)	PC2 (21.1%)
BPA	**+0.456**	**+0.210**
pH	+0.052	+0.222
Turbidity	–0.375	+0.096
DO	–0.450	–0.236
EC	+0.197	**+0.585**
Agriculture land-use proportion	–0.510	+0.079
Urban land-use proportion	+0.185	–0.517
Industrial land-use proportion	+0.172	–0.325
Industrial facility counts	+0.289	–0.347

Table S7. Hazard quotient (HQ) values of BPA exposure via drinking water across sampling points and population groups (adults and children), Related to result in part 'Human health risk assessment'

Sampling point	Adult		Children	
	Female	Male	Female	Male
SP 1	1.68E-05	1.92E-05	3.86E-05	3.77E-05
SP 2	1.95E-05	2.24E-05	4.49E-05	4.39E-05
SP 3	2.04E-05	2.33E-05	4.68E-05	4.57E-05
SP 4	1.56E-05	1.79E-05	3.59E-05	3.51E-05
SP 5	1.52E-05	1.75E-05	3.50E-05	3.43E-05
SP 6	2.27E-05	2.60E-05	5.22E-05	5.11E-05
SP 7	3.51E-05	4.01E-05	8.06E-05	7.88E-05
SP 8	3.70E-05	4.23E-05	8.50E-05	8.31E-05
**Average**	**2.28E-05**	**2.61E-05**	**5.24E-05**	**5.12E-05**
